# Association between Serum Vitamin D Concentration Status and Matrix Metalloproteinase-9 in Patients Undergoing Elective Percutaneous Coronary Intervention

**DOI:** 10.22037/ijpr.2020.112292.13670

**Published:** 2020

**Authors:** Farzaneh Foroughinia, Mahtabalsadat Mirjalili

**Affiliations:** *Department* of Clinical Pharmacy, Faculty of Pharmacy, Shiraz University of Medical Sciences, Shiraz, Iran.

**Keywords:** Vitamin D, Matrix Metalloproteinase-9, Percutaneous Coronary Intervention, Vitamin D deficiency, Cardiovascular diseases, Acute Coronary Syndrome

## Abstract

Cardiovascular diseases (CVD) have become increasingly life-threatening during recent decades. Several studies have shown that matrix metalloproteinase-9 (MMP-9) plays an important role in the process of atherosclerosis and heart remodeling. On the other hand, Vitamin D deficiency has been recognized as a risk factor for CVD. According to the prevalence of vitamin D deficiency in our country, Iran, we aimed to evaluate the relationship between vitamin D status and the level of MMP-9 in patients undergoing percutaneous coronary intervention. In this prospective cross-sectional study, the patients who were candidates for elective coronary angioplasty were included. Baseline serum MMP-9 and vitamin D levels were measured before intervention. The patients were categorized into three groups: Vitamin D-severely deficient (≤ 10 ng/mL), vitamin D-moderately deficient (11-20 ng/mL), and vitamin D-insufficient/sufficient (> 21 ng/mL). Totally, 150 patients were assessed. The analysis showed that serum MMP-9 levels were higher in patients with lower vitamin-D concentrations. A significant inverse correlation was found between MMP-9 concentration and 25 (OH) vitamin D level (*P* = 0.039). According to our results, it may be concluded that low levels of vitamin D may lead to more vulnerable atherosclerotic plaques and consequently more cardiovascular adverse effects in post-PCI patients.

## Introduction

Cardiovascular diseases (CVD) are one of the main causes of death worldwide and an important threat to human well-being in the twentieth century. According to third Sustainable Development Goal, preventing CVD can reduce one third of premature mortality due to noncommunicable diseases (1, 2). Atherosclerosis is defined as a progressive disease in which lipids and fibrous elements accumulate in the large arteries (3). This chronic inflammatory condition is the dominant cause of CVD including myocardial infraction (MI), stroke, heart failure, and claudication (4). Although atherosclerosis can induce CVD through arterial stenosis and reduction in blood flow, it is though that the major mechanism associated with CVD involves the inflammation which affects the stability of atherosclerotic plaques and facilitates their rupture (5). Studies have shown that agents with anti-inflammatory properties, such as omega-3 polyunsaturated fatty acids can be considered as an effective adjunctive therapy to the standard drug regimen used before PCI to decrease cardiovascular events after PCI (6, 7). Coronary plaque disruption followed by platelet aggregation and thrombosis, finally results in acute coronary syndromes and its complications (8).

Matrix metalloproteinases (MMPs), a family of zinc and calcium dependent endopeptidases, are known to have major role in cellular migration, cytokine activation, growth factor availability, angiogenesis, and extracellular matrix (ECM) degradation (9). MMPs, as well as other proteinases, trigger destruction of the vascular ECM in late stage atherosclerosis (10). MMPs have a dual role in intimal thickening which involves net matrix deposition, and plaque rupture, which involves net matrix destruction. Intimal thickening is defined as a physiological response to mechanical injury, increased wall stress, or chemical insult, such as atherosclerosis while plaque rupture is caused by remodeling failure of intima. It seems that the dysregulation of MMP activity mediates the transition from physiologic remodeling to matrix destruction (11). Among this family, MMP-9, also known as gelatinase B, is mainly responsible for the degradation of elastin and collagen IV and is produced by various cell types, including keratinocytes, monocytes, tissue macrophages, and polymorphonuclear leukocytes (neutrophils), as well as by a variety of malignant cells (12, 13). It has been demonstrated that serum MMP-9 levels are significantly elevated in ACS patients compared to the control subjects (14). Furthermore, mounting proof shows that MMPs play an important role in restenosis after angioplasty and the occlusion of venous artery bypass grafts through enhancing the proliferation and migration of endothelial and vascular smooth muscle cells (VSMC) (10, 15). 

The role of vitamin D in bone metabolism and maintenance of calcium hemostasis is well described (16). The deficiency of this fat soluble vitamin can lead to rickets and osteomalacia in pediatrics and adults, respectively (17). In recent years, it has been recognized that vitamin D deficiency is associated with several chronic medical conditions such as osteoporosis, cancers, autoimmune diseases, diabetes mellites, hypertension, and cardiovascular disease. Several studies suggest that the hormonal derivative of vitamin D, 1,25 dihydroxy vitamin D, directly affects cardiac muscle, regulates secretion of parathyroid hormone, regulates the renin angiotensin aldosterone system, and modulates the immune system (18). Although vitamin D deficiency is considered as an independent risk factor for CVD, it is not clear whether vitamin D supplementation can reduce the occurrence of CVD or improve its outcome (19).

There is evidence that suggests that vitamin D can positively affect MMP-9 serum level and reduces its production (20). With this background in mind, we aimed to evaluate the correlation between serum vitamin D concentration status and MMP-9, as an early cardiac remodeling biomarker, in the patients who were candidates for elective percutaneous coronary intervention.

## Experimental

In this prospective, cross-sectional study, 150 patients undergoing elective coronary intervention in a tertiary care heart hospital, Kosar hospital, affiliated to Shiraz University of Medical Sciences (SUMS) were evaluated during the study period, between January 2017 and July 2017. 

This study was approved by the Ethics Committee of SUMS. A written informed consent was obtained from all participants before enrollment to the study. 

Patients aged 18-80 years old, who has had a successful PCI and were willing to participate in the study were eligible to be enrolled. The exclusion criteria were as follows: administration of vitamin D supplement during last month, bypass surgery during three months prior to the study, STEMI patients, age above 80 or below 18 years old, and the patients unwilling to continue the study.

The patients’ demographic and clinical data, including sex, age, and risk factors for cardiac events such as history of smoking or past history of smoking, hypertension, hyperlipidemia, diabetes mellitus, myocardial infarction, acute coronary syndrome, percutaneous coronary intervention, and coronary artery bypass grafting (CABG) surgery were recorded.

Peripheral venous blood samples (10 mL) were collected from each patient before PCI, to determine 25-hydroxyvitamin D (25-OH D) and MMP-9 serum level. In order to separate serum from blood, the samples were centrifuged at 3,000 rpm for 10 min, then the samples were frozen and stored at -80 ℃ until measurement.

Serum level of vitamin D was measured by means of high-performance liquid chromatography (HPLC) while serum level of MMP-9 was determined by 96-well enzyme-linked immunosorbent assay (21) kit manufactured by Bioassay Technology Laboratory, Shanghai, China (E0936Hu).

Based on the objective of the study, the patients were stratified into three groups according to their vitamin D serum level as follows: Vitamin D-severely deficient (≤ 10 ng/mL), vitamin D-moderately deficient (11-20 ng/mL), and vitamin D-insufficient/sufficient (> 21 ng/mL) (18).

The data obtained were analyzed using the Statistical Package for Social Sciences (SPSS) (version 21 SPSS Inc., Chicago, Ill, USA). Categorical variables were presented as absolute and relative (percentage) frequencies. Continuous variables were expressed as mean ± standard deviation (SD). Due to the abnormal distribution of data, Kruskal-Wallis test was performed to examine the relationship between vitamin D level categories and serum concentration of MMP-9. Mann-Whitney test was also applied to determine any possible correlations between MMP-9 level and the patients’ gender. Pairwise comparison tests were conducted to determine which groups differ significantly from the other group by its MMP-9 level, using Mann-Whitney test. For all tests, *P*-value < 0.05 was considered as significance level.

## Results and Discussion

During the study period, a total number of 150 patients undergoing elective coronary intervention in Kosar hospital were recruited in the study. The average age of participants was 60.92 ± 10.07 years. The majority of the patients were male (57.3%). The mean serum vitamin D and MMP-9 levels were 17.40 ± 10.26 ng/mL and 3497.43 ± 3210.49 ng/mL, respectively. The participants’ demographic, clinical, and biochemical variables are reported in Table 1. 

Severe vitamin D deficiency was present in 37 (24.66%). Moderate vitamin D deficiency was reported in 66 (44%) of the participants while 66 patients (44%) had normal or insufficient vitamin D level. There was no significant difference regarding demographic and clinical characteristics amongst groups except for sex (*P*-value = 0.017) and previous history of PCI (*P*-value = 0.040). 

**Figure 1:**
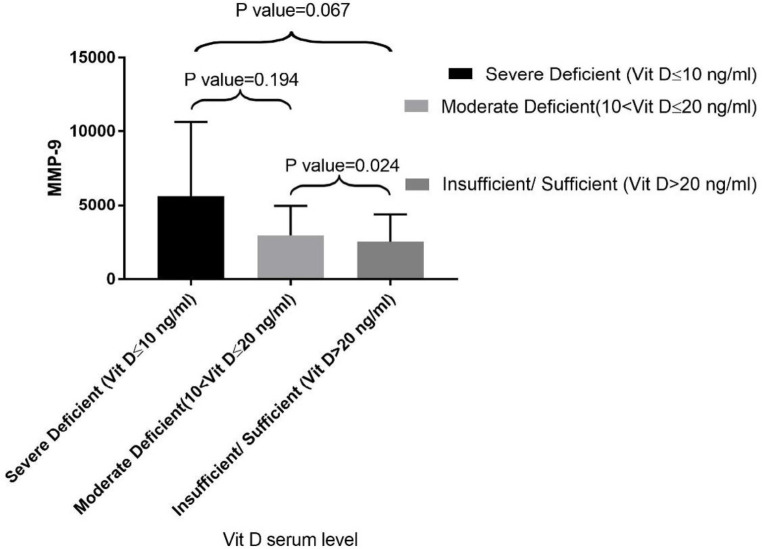
The association between serum vitamin D level with MMP-9

Further analysis showed no significant difference in the serum MMP-9 level in males and females (*P*-value = 0.682). The correlation of vitamin D level with MMP-9 is depicted in Figure 1 there was a significant inverse relationship between the level of vitamin D and the level of MMP-9 (*P*-value = 0.013). Evaluating the difference of MMP-9 level between three groups, it turned out that MMP-9 level was statistically different only between group 2 and group 3 (*P*-value = 9.024) but not between group 1 and 2 (*P*-value = 0.194) as well as group 1 and 3 (*P*-value = 0.067).

Our findings revealed that the serum concentration of vitamin D was low in the majority of the patients who were candidates for PCI. In other words, 103 out of 150 participants (approximately 70%) were suffering from severe or intermediate vitamin D deficiency. Several studies have proven that patients with serum level of 1, 25 dihydroxy vitamin D3 above 30 ng/mL, the cutoff defined as vitamin D sufficiency, are at lower risk of developing CVD (22, 23). Vitamin D deficiency is associated with arterial hypertension, obesity, impaired glucose metabolism and diabetes type 2, abnormal lipid profile, chronic kidney disease, endothelial dysfunction and atherosclerosis (24). Although the history of previous PCI was reported statistically significant between three vitamin D groups, this difference was seen between group 1 and group 3 and also group 1 and group 2. With regard to the definition of vitamin D deficiency (25 (OH) D < 20 ng/mL), there is no significant differences between vitamin D deficient patients and vitamin D sufficient cases based on this item. Based on the Framingham Offspring Study, severe vitamin D deficient patients (25 (OH) D < 10 ng/mL) who had never experienced CVD events, were more prone to develop a first CVD event after 5 years of follow-up compared to subjects with higher levels of vitamin D (25 (OH) D > 15 ng/mL) (25). 

A few randomized clinical studies have focused on the cardiovascular outcomes of vitamin D supplementation (24). Some studies failed to demonstrate any benefits of vitamin D supplementation on the prevalence of CVD or its mortality rate (26, 27). Also, there are metanalyses reporting non-significant trends for reduced cardiovascular events in the patients receiving vitamin D compared to the control groups (28, 29). On the other hand, the positive effect of vitamin D supplementation on the reduction of mortality rate has been reported in several other studies (28, 30). Therefore, the positive effect of administration of this supplement in the prevention of cardiovascular events has been remained controversial in recent years.

Our results indicate that the serum level of MMP-9 is inversely associated with vitamin D concentration among the patients undergoing PCI. This finding is consistent with the study of Khalili *et al*. in which an inverse relationship between 25 (OH) D level and MMP-9, as early biomarker of remodeling, was observed in the patients following myocardial infarction (31). In another study which was conducted on Iranian population, the association of vitamin D receptor gene polymorphism, serum levels of 25 (OH) vitamin D, and MMP-9 with coronary artery diseases (CAD) was evaluated. It was shown that vitamin D deficiency was associated with increase of serum MMP-9 level in the patients with coronary arterial disease. Moreover, the patients with ff genotype of FokI polymorphism had higher MMP-9 levels and lower vitamin D concentrations (32). Timms and his colleagues also demonstrated that vitamin D deficiency was associated with abnormal increases in circulating MMP-9 and MMP-2, as well as C-reactive protein (CRP). Interestingly, these abnormalities were corrected through receiving vitamin D supplementation for one year (33).

Furthermore, the impact of vitamin D level on the serum level of MMP-9 has been determined in other diseases, such as end stage renal disease, pulmonary tuberculosis, chronic rhinosinusitis with nasal polyposis, diabetic foot ulcer, etc. (34-37).

The exact mechanism behind the inhibition of MMP-9 production by vitamin D is unclear. It seems that activation of vitamin D receptor by its ligand can induce binding of activating protein-1 (AP-1) to its response elements existing on the MMP-9 gene promotors. For example, calcitriol or vitamin D3 increases the AP-1 binding activity and this process is dependent on binding of c-fos/c-jun elements vitamin D response element (VDRE) and AP-1 in the regulatory region of c-jun (38, 39). Also, vitamin D may inhibit MMP-9 production via suppressing tumor necrosis factor alpha (TNF-α) (37).

Several studies have shown that high level of MMP-9 is associated with higher risk of CVD, ACS, left ventricular remodeling post MI, and coronary artery in-stent restenosis (40, 41). Hence, serum MMP-9 level can be utilized as a specific and sensitive biomarker in clinical practice in identification of patients with high risk of CVD, diagnosis of atherosclerotic plaque rupture, and ischemia at early stages, and prevention of post MI remodeling (40).

Despite randomization, in our study, gender was statistically different between groups (*P*-value = 0.017). To remove the cofounding effect of gender, the Mann-Whitney test was used. Our analysis reported that the difference regarding gender distribution between the groups did not have any significant effect on serum level of MMP-9 (*P*-value = 0.68). In addition, it was revealed that most of our vitamin D deficient patients were male. With regard to the inverse relationship between vitamin D and MMP-9 levels in our results, it may be postulated that males with vitamin D deficiency are more prone to cardiovascular diseases than females. This finding is consistent with the Framingham heart study claiming that males with elevated plasma level of MMP-9 may be at greater risk for cardiac diseases, such as cardiac wall thickness and large end-diastolic left ventricular size (42). 

There were some limitations in the present study that should be considered: The major limitation was the relatively small sample size of the patients that has limited the statistical power. In addition, only one sample was taken from each patient and the patients were not followed up after PCI. Further studies with larger sample size considering measurement of serum MMP-9 and vitamin D level before and after PCI are required to confirm the impact of serum level of vitamin D and MMP-9 on CVD events and the outcomes of the treatment.

**Table 1:** Comparison between demographic and clinical characteristics of patients with severely deficient, moderately deficient and insufficient/sufficient vitamin D levels

	**Vitamin D3**			
***P*** **-value**	**Group 3** **(x > 20 ng/mL)**	**Group 2** **(10 < x ≤ 20 ng/mL)**	**Group 1** **(x** ** ≤** ** 10 ng/mL)**	**Variable**
	47 (31.3)	66 (44)	37 (24.7)	N (%)
0.017	20 (23)	45 (50)	24 (27)	Sex (male), N (%)
0.077	63.00 ± 8. 25	60.86 ± 11.91	58. 40 ± 7.58	Age, yrs, (mean ± SD)
0.175	17 (34)	17 (34)	16 (32)	Diabetes mellitus, N (%)
0.433	37 (35)	45 (42)	25 (23)	Hypertension, N (%)
0.587	21 (33)	30 (47)	13 (20)	Dyslipidemia, N (%)
0.212	6	16 (50)	10 (31)	Smoker, N (%)
0.376	3 (60)	2 (40)	0	Previous MI, N (%)
0.462	8 (22)	17 (49)	10 (29)	Previous smoker, N (%)
0.040	7 (58)	5 (42)	0	Previous PCI, N (%)
0.790	3 (43)	3 (43)	1 (14)	Previous CABG, N (%)
0.720	1 (20)	2 (40)	2 (40)	ACS, N (%)
0.103	65.55 ± 33.13	71.58 ± 29.14	79.61 ± 64.16	GFR, mL/min, mean ± SD
0.182	1.23 ± 1.03	1.08 ± 0.22	1.15 ± 0.28	Creatinine, mg/dL, mean ± SD

Abbreviations: MI, Myocardial infarction; PCI, Percutaneous coronary intervention; CABG, coronary artery bypass grafting; ACS, Acute coronary syndrome; GFR, Glomerular filtration rate.

## Conclusion

Given the importance of remodeling in the patients undergoing PCI, it can be suggested that identification and management of the patients with vitamin D deficiency prior to PCI, may prevent or decrease post-PCI cardiovascular adverse effects and in-stent restenosis. However, further studies are required to confirm the effect of vitamin D supplementation in preventing cardiovascular events after PCI. 
